# Retinal lesion annotation on fundus imaging: an interobserver variability study

**DOI:** 10.1038/s41598-026-53558-5

**Published:** 2026-05-28

**Authors:** Gabriel Lepetit-Aimon, Clément Playout, Marie Carole Boucher, Renaud Duval, Michael H. Brent, Frederic Lesage, Farida Cheriet

**Affiliations:** 1 Polytechnique Montréal, QC H3T 1J4 Montréal, Canada; 2 Département d’ophtalmologie, Université de Montréal, Faculté de Médecine, Montréal, Canada; 3 Hôpital Maisonneuve-Rosemont, Centre Universitaire d’ophtalmologie de l’Université de Montréal, Montréal, Canada; 4 University Health Network and Mount Sinai Hospital Toronto, Toronto, Canada

**Keywords:** Diabetic retinopathy, Retinal lesion segmentation, Interobserver variability, Fundus photography, Semi-automated annotation, Biomarkers, Computational biology and bioinformatics, Diseases, Health care, Medical research

## Abstract

Accurate detection and segmentation of retinal lesions in fundus photographs is essential for diagnosing diabetic retinopathy (DR) and for developing reliable machine learning models for this purpose. However, the reliability of manual annotation of retinal lesions by clinical experts has yet to be evaluated. We quantified interobserver variability in the pixel-wise annotation of microaneurysms (µA), intraretinal hemorrhages (Hem), hard exudates (Ex), and cotton-wool spots (CWS) using 51 images from the MAPLES-DR dataset, independently annotated by three senior retinologists. Manual segmentation showed substantial variability: 38% of lesions marked by one observer were undetected by the others, lesion coordinates differed by approximately one third of the lesion diameter, and the mean interobserver IoU was 0.47, indicating inconsistent contour delineation even when observers detected the same lesion. Paradoxically, inter-expert DR grading assessments were largely concordant, suggesting reliance on more holistic or experience-based judgment criteria rather than on lesion counting. We assessed a semi-automated workflow in which annotators corrected model-generated pre-segmentations. This approach markedly improved agreement, yielding a pixel-wise mean IoU of 0.71, reducing both detection and contour-related variability; over 90% of lesions were preserved directly from the pre-segmentation.

## Introduction

Diabetic retinopathy (DR) is a complication of diabetes mellitus characterized by damage to the retinal microvasculature, which can lead to vision impairment. In 2020, DR affected approximately 22% of people with diabetes–an estimated 103.12 million adults worldwide–making it the leading cause of blindness among adults aged 25 to 75 years^1,2^. Due to its gradual progression, DR is routinely monitored in at-risk populations through large-scale screening programs based on non-invasive fundus imaging^3–5^. These programs follow international guidelines that associate clinical management pathways with severity grades defined by the number and type of retinal lesions^6–8^.

Microaneurysms, hard exudates, and cotton wool spots are typically associated with low severity grades; while intra-retinal hemorrhages indicate mild diabetic retinopathy (DR) when few in number or severe DR if they are more numerous. In the pursuit of developing automatic and interpretable DR grading algorithms, multiple methods have been proposed to automatically segment retinal lesions^9–11^, supported by the publications of several datasets over the last decade^12–17^. Recently, our team has released the MAPLES-DR dataset^18^: a set of 198 fundus images paired with expert pixel-wise annotations of the anatomical and pathological structures of the retina, including those four lesions. The variability throughout the course of the annotation process prompted us to question its reliability and the consistency with which clinical experts label retinal lesions at the pixel level.

Interobserver variability is well documented among senior and junior retinologists performing DR grading^19,20^. However, to our knowledge, no study has investigated such variability at the level of the lesions that inform those grades. Prior work on segmentation consistency has focused on anatomical structures, revealing substantial interobserver differences in the manual delineation of retinal vessels^21,22^ as well as the optic disk or cup^23,24^. Fundus lesions pose an even greater challenge: they are less contrasted, span a wider range of sizes and shapes, and their detection and classification rely more heavily on subjective interpretation. While inconsistencies in lesion-level annotations are therefore expected, they have not been quantitatively characterized prior to this work.

Characterizing interobserver variability in retinal lesion annotation serves two complementary purposes. Clinically, understanding disagreement at the lesion level may help explain variability in DR grading and inform refinements of grading standards to improve their robustness. Methodologically, manual lesion annotations are used as ground truth for training and evaluating machine-learning models; variability in these annotations directly constrains both achievable performance and the reliability of evaluation metrics. Quantifying this variability is therefore essential to choose algorithms and validation strategies that are less sensitive to interobserver disagreement and better reflect the intrinsic uncertainty of manual lesion segmentation.

A further motivation for this study concerns annotation workflows. Because manual lesion labeling is time-consuming, several datasets rely on automated pre-segmentation to initialize annotations that are subsequently reviewed and corrected by experts. This strategy was adopted, for example, in the construction of DDR^15^ and MAPLES-DR. However, despite its growing use, the impact of pre-segmentation on interobserver consistency–and the potential biases it may introduce–has not, to our knowledge, been systematically evaluated.

In this work, we systematically investigate interobserver variability in the pixel-wise annotation of microaneurysms (*μ*A), intraretinal hemorrhages (Hem), hard exudates (Ex), and cotton-wool spots (CWS). Three senior retinal specialists independently annotated these lesion types in 51 fundus images from the MAPLES-DR dataset, enabling a detailed comparison of manual segmentations. A second annotation phase was then conducted on a subset of images in which the clinicians reviewed and corrected AI-generated pre-segmentation maps.

Our contributions are threefold. First, we provide the first comprehensive quantification of interobserver variability in fundus lesion segmentation, explicitly disentangling variability arising from lesion detection, lesion classification, and boundary delineation, and quantifying its impact on DR diagnostics. Second, we assess the benefit of AI-generated pre-segmentation maps to reduce interobserver variability during annotation. Third, we derive practical recommendations for the annotation and curation of retinal lesion ground truths, as well as the evaluation and validation of segmentation models.

## Materials & methods

### Data selection

**Fig. 1:**
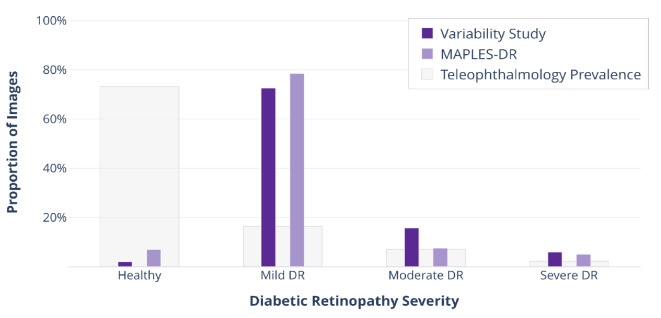
The majority of the images analyzed in this study show signs of mild diabetic retinopathy (DR). This biais reflects the prevalence observed in tele-ophthalmology screening programs^25^ (healthy cases excluded).

The 51 fundus images analyzed in this study were selected from those annotated by our team during the construction of the MAPLES-DR dataset and originate from the publicly available MESSIDOR database. All selected images exhibit signs of diabetic retinopathy, and 73% of them were diagnosed as mild DR (see Fig. 1). This focus on mild DR is inherited from the MAPLES-DR dataset and reflects the prevalence observed in clinical screening for DR via tele-ophthalmology, once healthy cases are excluded. MESSIDOR retinographs were acquired with a Topcon TRC NW6 non-mydriatic camera and have a 45 degree field of view in high resolution. In addition, all images are well illuminated, well focused, and have been rescaled to *1500× 1500* pixels prior to the annotation campaign.

### Study design

This study was conducted in two phases. We first quantified interobserver variability when manually segmenting lesions. We then evaluated how providing AI-generated pre-segmentation maps biased the annotators’ annotations and impacted interobserver variability.

### Phase I: Annotation without pre-segmentation

In the first phase of this study, three senior retina specialists–referred to as S1, S2, and S3–were asked to manually annotate the four lesion types. They were chosen for their past involvement in Canadian teleophthalmology screening programs. All were experienced with the Canadian standard for DR screening^8^ and participated in the annotation of MAPLES-DR two years earlier. Given this time interval, annotators were likely to have forgotten their previous annotations but would still be familiar with the annotation tool. No specific guidelines were provided to the retina specialists prior to the annotation campaign, apart from the list of lesion types to be segmented. In particular, no instructions were given regarding the expected level of annotation precision, tool settings, zoom level, or choice of preprocessing for the fundus images. Such guidelines might have reduced interobserver variability; however, our objective was to explore interobserver variability across all its facets, including biases in annotation granularity. The labeling process was conducted on the web-based annotation platform described previously in the MAPLES-DR publication^18^. All retina specialists were familiar with this tool, especially its visualization enhancement features (fundus pre-processing and contrast maximization).

### Phase II: Annotation assisted by pre-segmentation

For the second phase of this study, one of the senior retina specialists (S1) and two junior retinologists (J1 and J2) were asked to annotate 16 fundus images again from those used in the first phase, but this time assisted by AI-generated pre-segmentation maps. S2 and S3 were not available to participate in this second phase. All annotators were provided with the same initial maps, which were generated using a U-Net with a ResNeXt50 as the encoder, trained on IDRiD^14^, DDR^15^, and FGADR^16^. We released the code and weights of the model as a standalone library named fundus-lesions-toolkit, which is publicly available on github^26^. Similarly to the annotation process of MAPLES-DR, annotators had to review the lesions detected by the model, erase the erroneous ones, and then manually delineate lesions that were missing from the pre-segmentation maps.

## Results

### Phase I: Interobserver variability for manual annotation

As a first estimate of the interobserver variability in manual annotation of retinal lesions, we compared the segmentation maps produced by the three senior retina specialists using the mean Intersection over Union (mIoU). This metric is widely used to evaluate fundus lesion segmentation pixel-wise, as it accommodates the inherent sparsity of lesion segmentation maps and condenses per-lesion IoU scores into a single average value. In the context of this study, the IoU offers the additional advantage of considering both segmentation maps symmetrically, without designating either as a reference or ground truth.

**Fig. 2:**
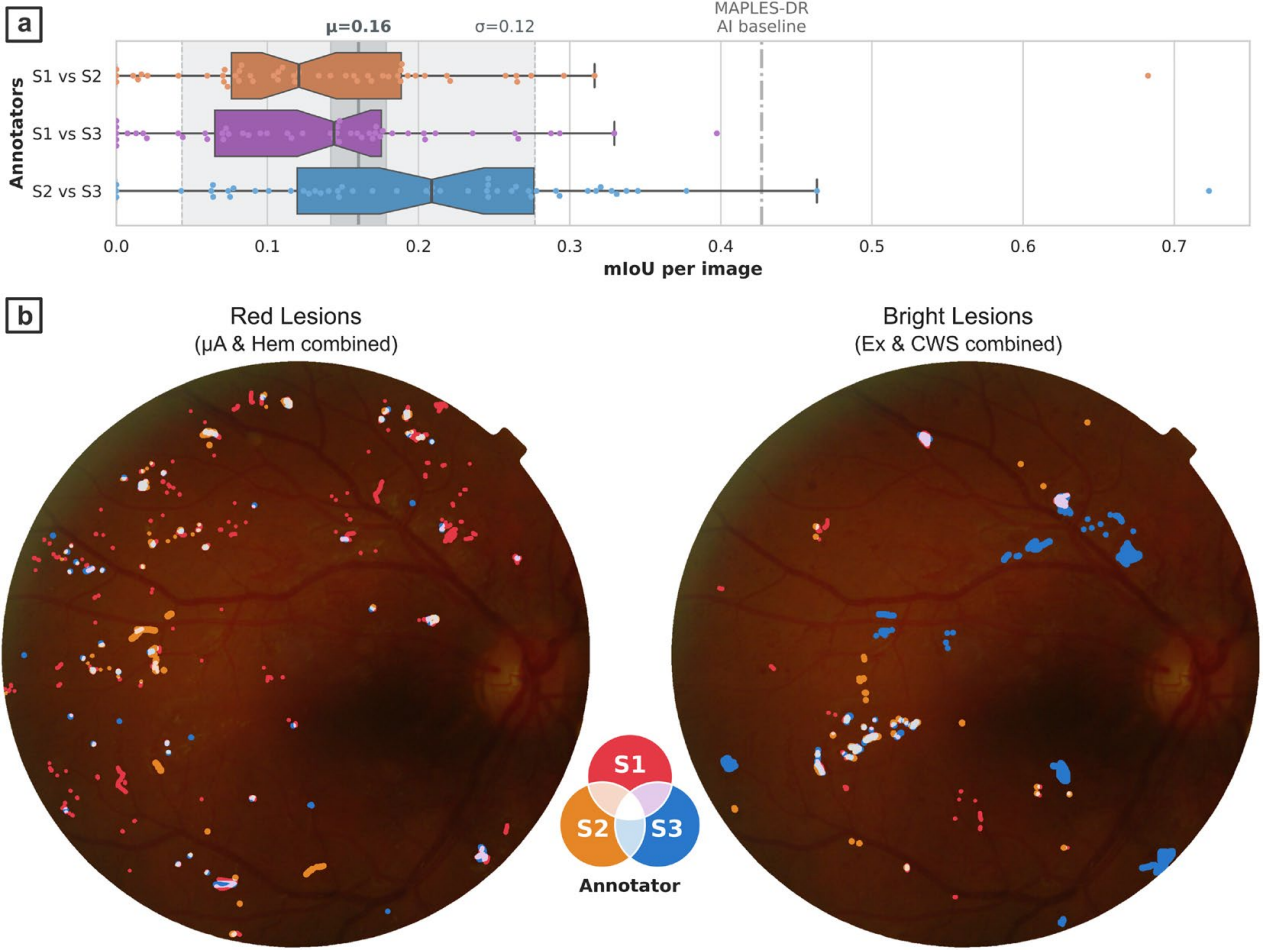
Senior retinologists manually labeling retinal lesions only agree on 16% of annotated pixels. **(a)** Pairwise pixel-wise agreement between annotators: mIoU is presented per image and averages the IoU of each lesion type. The mean and standard deviation in grey aggregates all three annotator pairs. The AI baseline providing the reference mIoU was respectively trained and evaluated on MAPLES-DR train and test sets which include the 51 images of this study. **(b)** Qualitative example of disagreements for manual lesion annotation. Plain colors encode pixels only annotated by a single annotator, light colors indicate partial agreement between two annotators, light grey indicates complete agreement.

Across all pairs of annotators, the mean mIoU is 0.16 (SD=0.12, Fig. 2a). The agreement rate varies substantially from one image to another; half of the per-image mIoU values range between 0.075 and 0.219. A similar trend is observed across all annotators, despite a significantly higher agreement between S2 and S3 compared to pairs involving S1 (*p<.001* for the Wilcoxon signed-rank test). Such levels of agreement are substantially lower than expected. For comparison, the top 4 models submitted to the IDRiD segmentation challenge achieved mIoU scores between 0.55 and 0.65; our own rudimentary baseline model trained and tested on MAPLES-DR images reached an mIoU of 0.43. Three-quarters of the agreement rates measured in our study do not reach half of this baseline score. In other words, automatic segmentation models appear to reproduce expert annotations more closely than the experts themselves do.

A brief qualitative examination of the differences between the manual annotation of our three senior retina specialists (Fig. 2b) reveals three causes for these low agreement rates. **(1)** The exhaustiveness and granularity of the annotations vary among annotators: S1 annotates more red lesions than S2 or S3, and S3 segments the bright lesions more coarsely than the other two; **(2)** Many lesions were detected by only one or two annotators and rarely by all three; **(3)** Among the lesions that were indeed identified by two or three annotators, the delineation of their boundaries differs from one retinologist to another.

The following sections explore the different components of the interobserver variability affecting the manual annotation of retinal lesions–namely, the variability in granularity, detection, classification, and finally segmentation.

### Discrepancies in annotation granularity

**Fig. 3:**
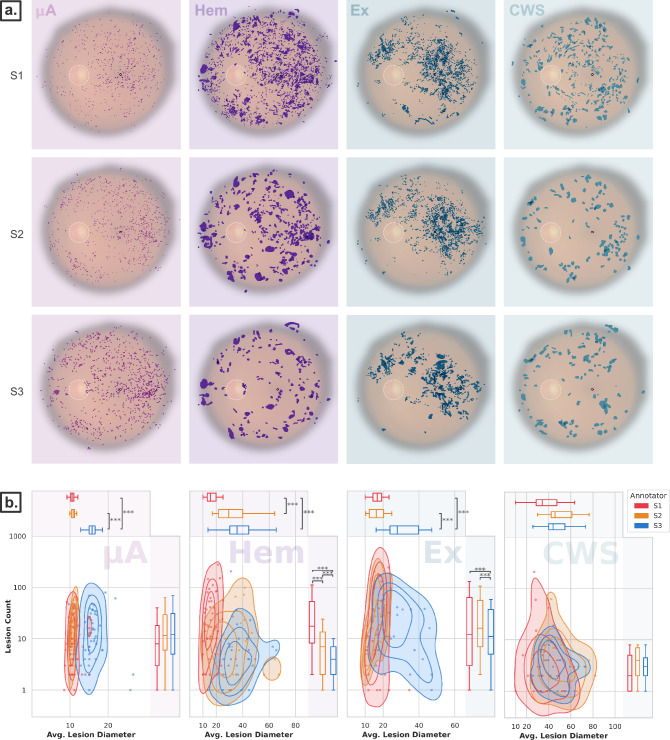
Manual annotation style differs among retina specialists inducing significant disparities in number and size of individual lesions annotated per image. **(a)** Unified representation of all annotated lesions over the dataset. All the images and their corresponding lesion maps were spatially registered on their macula and optic disk center, and then fused together into lesion atlases by annotator and lesion type. **(b)** Bivariate distributions of exhaustivity (count) and granularity (size) by lesion type and annotator. Each dot encodes the number of individual lesions and their average diameter for a single image. Diameters are derived from lesion areas by assuming that their shapes are circular. Significance is measured by two-sided Wilcoxon signed-rank test (***: *p<0.001*).

Each retina specialist exhibits a personal annotation style, making their segmentations almost identifiable from one another. Their styles differ in two main aspects: annotation exhaustiveness and acuteness in delineating lesion boundaries, which we will refer to as granularity. The unified representation of every annotated lesion–obtained by aligning all segmentation maps using the optic disk and macula as reference landmarks for registration–offers a qualitative overview of each clinician annotation style (Fig. 3a). Annotator S1 consistently annotated more lesions and used finer boundaries, whereas S3 segmented broader pathological regions, resulting in fewer but larger individual lesions. S2 exhibited intermediate behavior. This representation also highlights the concentration of lesions in the macular region, which is both the central area of the image and the most clinically significant. A stratified analysis comparing non-referable images (healthy and mild DR) with referable images (moderate and severe DR) corroborates the expected result: on average, retina specialists detect more lesions per image in patients with advanced DR, while the mean lesion diameter remains unchanged (see Supplementary Material).

Quantitatively, the differences in annotation granularity manifest as biases and variabilities in the number of individual lesions annotated per image and in their mean diameter, which depend on the lesion type (Fig. 3b). *Cotton Wool Spots* are annotated by all retina specialists as large regions and are relatively rare–fewer than ten lesions per image, with an average diameter of 44 pixels. For *microaneurysms* and *exudates*, the median number of lesions per image is consistent across annotators, but S1 and S2 delineate them significantly more precisely than S3. Regarding *hemorrhages*, S1 identifies four times more per image than S2 or S3, and with finer granularity.

Followup interviews with the clinicians revealed two external sources for those biases, in addition to the discrepancies in personal annotation style. **(1)** S1 more frequently activated the contrast-enhancement preprocessing when annotating red lesions, resulting in a significantly higher sensitivity to hemorrhages. **(2)** The default configuration of the annotation tool–a circular brush with a diameter of 10 pixels–explains the high concentration of lesions segmented with precisely that size. Although the tool allowed for a reduction in its diameter, the default configuration served as a practical lower bound for the annotation granularity for S1 and S2.

Beyond the variability in annotation granularity, the differences in exhaustiveness revealed by this first quantitative analysis point to deeper disagreements in lesion detection, which are investigated in the next section.

### Variability in lesion detection

Some lesions identified by an annotator do not overlap with any of the lesions segmented by others. Such discrepancies should not be interpreted as disagreements in *segmentation* but rather as disagreements in *detection*–that is, whether a pathological structure is deemed present or absent in a given region. Accordingly, the analysis of detection variability presented in this section is conducted not at the *pixel* level but at the level of each *lesion*, defined as a connected component computed from the segmentation maps. A lesion is considered jointly detected by two annotators if their respective segmentations overlap. However, because lesion size varies substantially among retina specialists–reflecting the differences in annotation granularity described earlier–a single lesion from one annotator may overlap with multiple lesions from another. For this reason, the analysis must be carried out relative to each annotator and subsequently averaged.

**Fig. 4:**
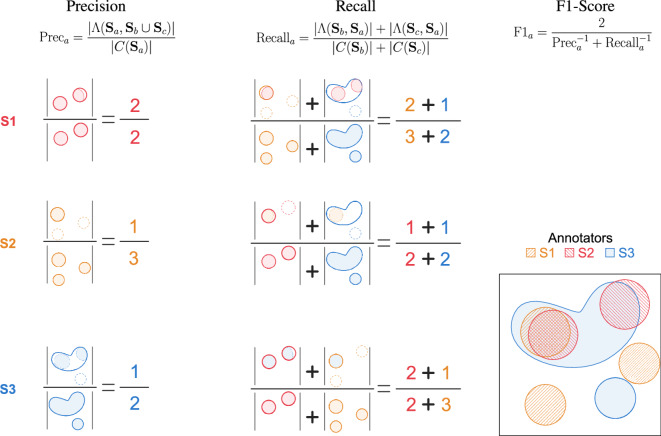
Definition and example of the custom precision and recall score used to compare three segmentation maps without ground truth. For a given annotator *a*, $\text {Prec}_a$ is the proportion of lesion that were also detected by any of the other two, and $\text {Recall}_a$ is the proportion of lesion also annotated by *a* among those annotated by the other two.

Overall, 38% (SD 17%) of all annotated lesions were identified by a single annotator and disputed by the other two; this corresponds to roughly one quarter of all annotated pixels. However, this general tendency masks substantial disparities across annotators and lesion types, which calls for a more detailed analysis. In the absence of a consensual ground truth we adapt the precision and recall formulas to quantify detection agreements between each annotator relative to the other two (Fig. 4).

Let ${\textbf {S}}\in \{0,1\}^{H\times W}$ be a lesion segmentation map, and $C({\textbf {S}})$ denote the set of individual lesions in ${\textbf {S}}$ (i.e. any connected component other than the background). We define $\Lambda \big ({\textbf {S}},\,{\textbf {S}}_{ref}\big )$ as the set of lesions in ${\textbf {S}}$ that are jointly detected in a second segmentation map ${\textbf {S}}_{ref}$. Using this notation, we derive three lesion-level detection metrics to quantify the agreement of annotator *a* relative to annotators *b* and *c*. The detection *precision* $\text {Prec}_a$ corresponds to the proportion of lesions annotated by *a* that are jointly detected in the segmentation maps of *b* or *c*, whereas the detection *recall* $\text {Recall}_a$ measures the proportion of lesions annotated by *b* or *c* that are jointly detected by *a*. Unlike the traditional definitions of precision and recall, these metrics are not computed with respect to a common ground truth: precision is defined relative to the lesions annotated by *a*, while recall is defined relative to the lesions annotated by the other annotators. Finally, the detection *F1-score* $\text {F1}_a$ is computed as the harmonic mean of these two quantities.

**Fig. 5:**
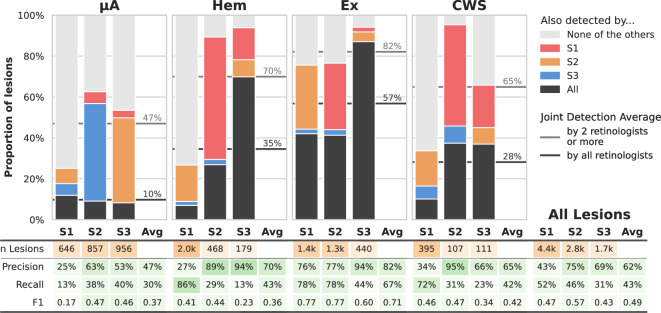
Out of all lesions manually annotated by each of the three senior retinologists, only 62% were detected by at least two annotators. Each bar encodes, among all lesions identified by a given annotator, the proportion which were also detected by none, one or two of the other annotators. A lesion is considered jointly detected by another retinologist if at least one of its pixels is also present in the segmentation map. Because the bar heights are normalized by the total number of lesions segmented by a given annotator–which differs from one to another–the apparent proportion of lesions jointly detected by all three varies greatly between annotators.

Across all lesion types, S1 had the lowest detection precision and the highest recall (Fig. 5). These differences reflect S1’s more exhaustive annotation style: S1 alone detected as many lesions as S2 and S3 combined. This annotation bias, particularly evident for hemorrhages and CWS, lowers S1’s detection precision and conversely increases that of S2 and S3, since their annotated lesions are more likely to appear in S1’s segmentation map. Yet, despite this effect, a third of the lesions annotated by either S2 or S3 were identified only by one of them. Overall, S2’s annotation style–detailed for microaneurysms and exudates but coarse for hemorrhages and cotton wool spots–proved to be the most consensual, achieving a per-image F1 score of 0.48, which is higher than that of S1 and S3 (0.38 and 0.41, respectively; 95% CI ±0.05 for all).

Interobserver agreement also varies substantially across lesion types. Hard exudates show the highest mean F1-score (0.71), with 57% jointly detected by all three retinologists. Although S3’s coarse annotation style inflates precision (94%), S1 and S2 still agree on 74% of the exudates they segmented. Cotton Wool Spots present the second-highest agreement (F1 = 0.42); their rarity and diffuse appearance likely contribute to increased variability. In contrast, red lesions show the lowest agreement, with comparable F1-scores for microaneurysms and hemorrhages (0.37 and 0.36).

Variability in microaneurysm detection is particularly concerning. Unlike hemorrhages–for which annotation styles differ substantially–microaneurysm counts remain consistent across annotators; yet only 10% are jointly detected by all three, compared with 35% of hemorrhages. Their small size, early pathological onset, and low contrast likely underlie this difficulty. Post-annotation interviews revealed that several microaneurysms segmented by S1 or S3 were, in fact, dust on the camera lens, consistently appearing across different patients at identical coordinates. Using this criterion, 1767 artifacts potentially confusable with microaneurysms were identified in 638 images from the MESSIDOR dataset. The interviews also highlighted recurrent disagreements in the classification of red lesions between microaneurysms and hemorrhages, which artificially lower their respective detection agreement rates and are examined in the next section.

Severe lesions (e.g., hemorrhages, exudates, and CWS) are detected more consistently in patients with advanced DR, likely because they appear less ambiguous in such images (see Supplementary Material). Surprisingly, however, microaneurysm detection exhibits higher interobserver variability in patients with advanced DR This may be because, in images with a higher overall lesion burden, microaneurysms receive comparatively less attention.

**Fig. 6:**
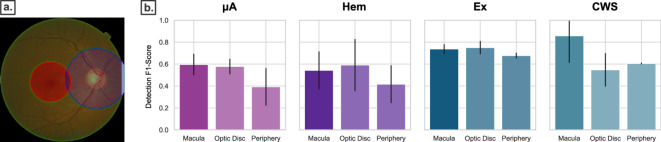
Interobserver variability in lesion detection is higher in the periphery than near the optic nerve head or the macula. **(a)** The optic nerve head is defined as a circular region around the Optic disk (OD) center, with three times the OD radius; the macular area is centered on the fovea with twice the OD radius; the peripheral region is everywhere else. The fovea and optic disk (OD) localizations and radius are extracted from the MAPLES-DR annotations. **(b)** Detection F1-Score by lesions and retinal regions, averaged on annotator pairs and images. Error bar indicates standard deviation.

Finally, we investigate whether the variability is conditioned on the region of the image being examined. We defined three regions of interest: the macula, the optic nerve head, and the peripheral retina (Fig. 6). We then assessed inter-annotator agreement within each of these zones. On average, agreement was highest in the macular region, which not only occupies the central part of the image but also corresponds to the area responsible for high-acuity central vision. Its clinical importance likely contributes to the increased attention and consistency observed among annotators. Clinicians may also struggle to consistently distinguish between hemorrhages and microaneurysms in peripheral areas due to reduced image quality, lesion size, and contrast in these regions.

### Variability in red lesion classification

Small hemorrhages and microaneurysms often exhibit similar appearances on color fundus photographs, and the nature of small red lesions is often unclear. This ambiguity drives substantial interobserver variability in classification: half of the microaneurysms segmented by one annotator are identified as hemorrhages by at least one of the others. These disagreements account for a significant share of the detection variability reported in the previous section. When considered separately, microaneurysms and hemorrhages exhibit low detection F1-scores (0.37 and 0.36) and rare consensual detection (10% and 35%); however, when treated as a single *red lesion* category, the interobserver consistency rises as the F1-score reaches 0.63, and all three clinicians jointly detect 52% of these lesions. Therefore, characterizing interobserver variability for red lesions requires a close examination of the consistency with which annotators distinguish microaneurysms from hemorrhages.

To quantify classification agreement, each red lesion segmented by annotator *a* and jointly detected by annotator *b* is assigned to one of three subsets: $\Lambda ^{\text {Hem}}_{ab}$ for lesions classified as both hemorrhages, $\Lambda ^{\mu \text {A}}$ for lesions classified as both microaneurysms, and $\Lambda ^{\text {Dis}}$ for lesions on which the annotators disagree. Formally:

$$\begin{aligned} \Lambda ^\text {Hem}_{ab}&= \Lambda \big ({\textbf {S}}_a^\text {Hem},\,{\textbf {S}}_b^\text {Hem}\big )&\Lambda ^{\mu \text {A}}_{ab}&= \Lambda \big ({\textbf {S}}_a^{\mu \text {A}},\,{\textbf {S}}_b^{\mu \text {A}}\big )&\Lambda ^\text {Dis}_{ab}&= \Lambda \big ({\textbf {S}}_a^\text {Hem},\,{\textbf {S}}_b^{\mu \text {A}} \setminus {\textbf {S}}_b^{\text {Hem}}\big ) \cup \Lambda \big ({\textbf {S}}_a^{\mu \text {A}},\,{\textbf {S}}_b^\text {Hem} \setminus {\textbf {S}}_b^{\mu \text {A}}\big ) \end{aligned}$$

The classification accuracy of red lesions segmented by *a*, relative to *b*, is then defined as:

$$\begin{aligned} \text {acc}_{ab} = \dfrac{ |\Lambda ^\text {Hem}_{ab}| + |\Lambda ^{\mu \text {A}}_{ab}| }{ |\Lambda ^\text {Dis}_{ab}| + |\Lambda ^\text {Hem}_{ab}| + |\Lambda ^{\mu \text {A}}_{ab}| } \end{aligned}$$

**Fig. 7:**
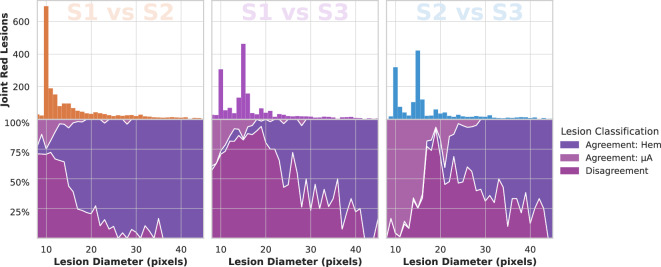
Senior retinologists disagree on the classification of 45% of small red lesions (diameter < 20 px) into microaneurysms versus hemorrhages. **(upper)** Number of red lesions jointly detected by two annotators per lesion diameters. Microaneurysms and hemorrhages are aggregated together. **(lower)** Proportion of classification agreement or disagreement amongst jointly detected red lesions.

Across annotator pairs, the overall classification accuracy is 51%. Discrepancies predominantly concern *small* lesions: 86% of all disagreements involve lesions <20 pixels in diameter. Within this size range, classification accuracy is 45%, increasing to 60% for lesions between 20–30 pixels and to 85% for larger lesions (Fig. 7). As fluorescein angiography is the clinical reference for differentiating microaneurysms from hemorrhages, reliance on color fundus images alone inevitably introduces uncertainty, with inconsistent classification even intraobserver.

Beyond visual ambiguity, systematic interobserver biases also contribute to classification variability. Annotator S1 classified most red lesions as hemorrhages (61% on average per image), whereas S2 and S3 predominantly labeled them as microaneurysms (77% and 88%, respectively; 95% CI ±8%). This divergence was accentuated for lesions on which annotators disagreed: among these, S1 labeled 76% as hemorrhages, compared with 30% for S2 and 6% for S3. We initially hypothesized that S1’s use of contrast-enhancement preprocessing, along with increased detection exhaustiveness, contributed to this difference. However, a joint review of discordant cases by S1 and S2 showed that their classification choices remained unchanged even without preprocessing, and despite adjudication, they could not agree on a common classification. These findings indicate that the observed variability reflects persistent differences in the interpretation of small red lesions, even among experienced retina specialists.

### Interobserver variability of lesion detection and DR grading

Standardized diabetic retinopathy (DR) screening systems rely on rule-based criteria that define disease severity according to the number and localization of specific pathological features, including those studied in this work. The interobserver variability documented in the previous sections raises concerns about the robustness of these rule-based frameworks. In particular, interobserver biases in lesion count or lesion type may propagate directly into DR grades, potentially leading to over- or under-diagnosis. To quantify the extent of this propagation, we compare *reference* DR grades against *rule-based* ones that are automatically deduced from the lesion segmentation maps.

**Fig. 8:**
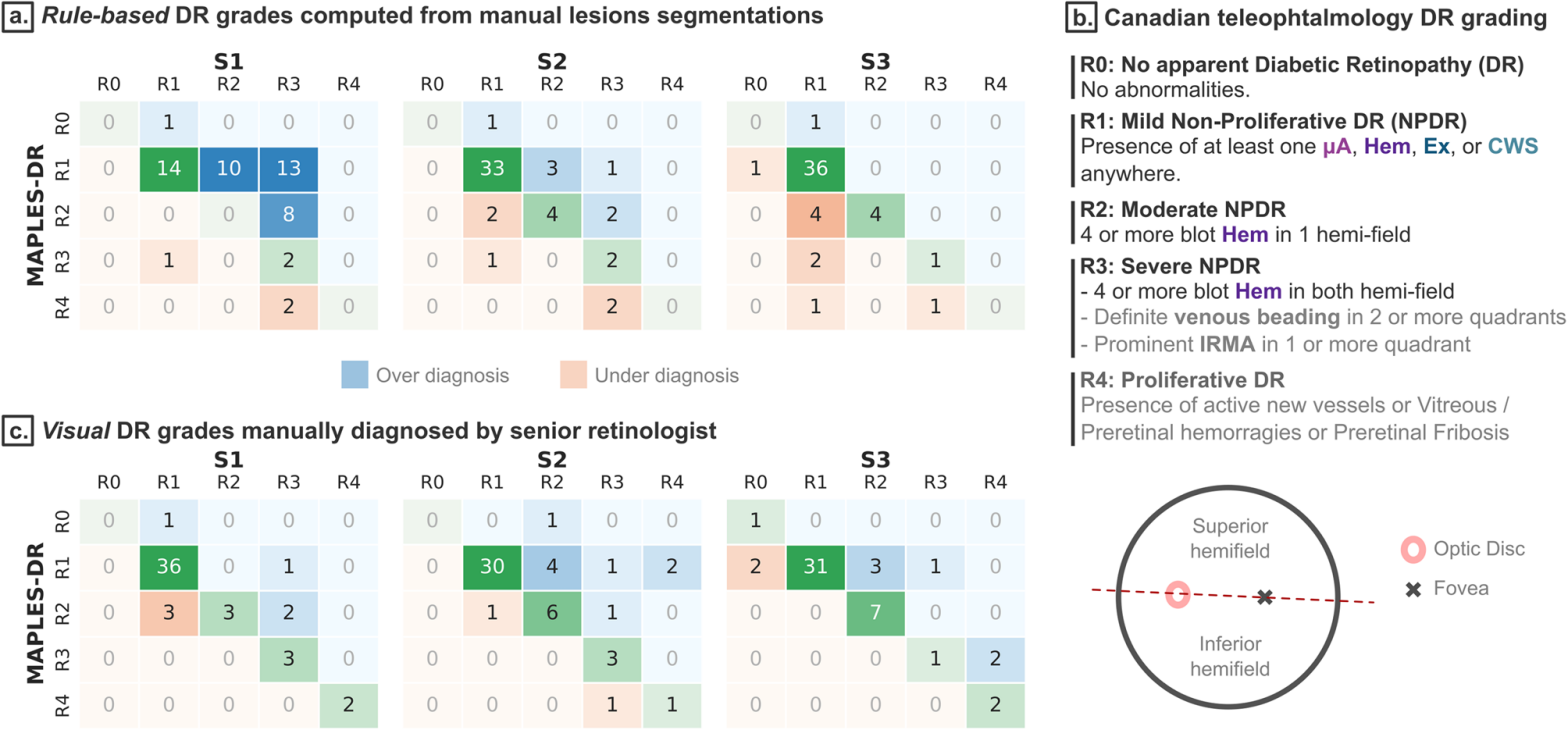
Rule-based DR grading systems are sensitive to lesion detection and classification biases–S1 mostly overdiagnoses (32/51 images, 63%), S3 tends to underdiagnose (9/51, 18%). **(a)** DR grades computed automatically from the lesions segmented by each retinologist and compared to MAPLES-DR ground truth labels. The grades are derived from a simplified version of the canadian teleophthalmology guidelines for Diabetic Retinopathy assessment^8^, summarized in **(b)**. Ignored criteria are in grey. **(c)** DR grades manually established by each retinologist under teleophthalmological conditions (using only a single fundus image) and compared to MAPLES-DR ground truth labels. The ground truth labels aggregate the individual manual grades by majority voting and advocacy.

For this comparison, the *reference* grades are the ones provided in MAPLES-DR–annotated by S1, S2, and S3 during the first annotation campaign and aggregated into a single ground truth label through majority voting and adjudication. Those grades follow the guidelines of the Canadian teleophthalmology screening program (summarized in Fig. 8b), whose rules were used to deduce the *rule-based* grades from the segmentation maps of each annotator. Because the rules defining the most severe stages rely on lesion types not segmented in this study (venous beading, IRMA, neovascularization), only criteria involving *μ*A, Hem, Ex, or CWS were considered. This simplification leaves stages R0 to R2 unchanged but restricts the definition of R3 and excludes the proliferative stage R4 from the rule-based grades; however, this should have a minimal influence on the results, as these two latter stages collectively account for only five of the 51 images analyzed. Indeed, according to MAPLES-DR reference grades, the majority of images are R1 (37/51, 72%) or R2 (8/51, 16%).

The comparison between rule-based and reference grades highlights how sensitive the grading rules are to segmentation biases (Fig. 8a). S1’s exhaustive annotation style results in a consistent over-grading of R1 and R2 images: 31 of 45 cases (69%) are classified as R2 or R3. Conversely, S3’s more conservative segmentation style results in half of the R2 and R3 images being underestimated as R1 (6 of 11 cases, 54%). In both scenarios, the determining factor is the number of hemorrhages–inflated for S1 and reduced for S3–which drives the diagnosis upward or downward. Hence, biases in the number of detected red lesions and in the tendency to classify them as hemorrhages rather than microaneurysms induce substantial drift in DR grading; at least when grading rules are applied literally.

Interestingly, when the same clinicians diagnose DR by simply reading the fundus image without manually annotating every lesion, the segmentation-related biases vanish (Fig. 8c). Under those conditions–mimicking real-world teleophthalmology screening–S1 becomes the least likely to overestimate DR severity and the most likely to underestimate it, reversing the pattern observed for lesion segmentation. Beyond annotation biases, interobserver variability is substantially higher when diagnoses are derived from segmentation maps than when assigned visually: the Gwet AC2 agreement coefficient reaches only 0.74 (95% CI 0.65–0.83) for rule-based grades, compared with 0.92 (95% CI 0.87–0.97) for visual grading. The discrepancies between rule-based and visually assigned grades indicate that clinical decision-making extends beyond the literal application of grading criteria and incorporates a substantial degree of expert subjectivity.

### Variability in lesion segmentation

Once disagreements in lesion detection and classification are excluded, the remaining discrepancies pertain solely to boundary delineation–that is, pure segmentation interobserver variability. Because it does not affect the number of annotated lesions, this source of variability does not influence rule-based DR grading. Yet, it can substantially impact segmentation models, whose training and evaluation typically rely on pixel-level loss functions and metrics. To isolate the variability due to segmentation disagreement only, we restrict the analysis to lesions jointly detected by at least two annotators, erasing all others from the segmentation maps. On those simplified maps, the global mIoU reaches 0.47 (95% CI ±0.014). In other words, even after adjusting for misdetection, fewer than half of the segmented pixels are shared between annotators.

This substantial segmentation variability manifests in two principal ways: discrepancies in the *localization* of the lesion and differences in its *shape* and *extent*. Regarding localization, visual inspection indicates that segmentations of the same lesion by different annotators are rarely concentric. On average, the distance between the centroids of two segmentations of the same lesion is 4 pixels for microaneurysms and 7 pixels for other lesion types, regardless of the annotator pair. These offsets correspond to approximately one third of the average lesion diameter.

**Fig. 9:**
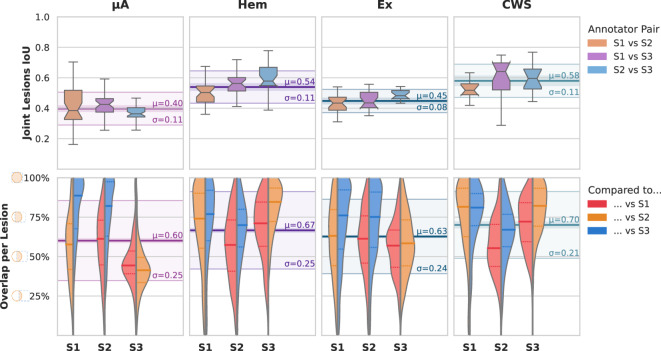
The segmentation of lesions jointly detected by two annotators differs by half of their pixels. **(upper)** IoU computed on segmentation maps containing only jointly detected lesions. Mean and standard deviation per lesion aggregate IoU over all images and annotators. **(lower)** Distribution of overlapping areas for each lesion segmented by an annotator and compared to another. Only jointly detected lesions are considered. Median and quartiles are indicated as plain and dotted lines.

Shape-related discrepancies further contribute to segmentation variability. As previously shown, the average lesion size differs across annotators. These differences directly influence the proportion of overlapping pixels: because S3 tends to segment microaneurysms and exudates more broadly than S1 and S2, most pixels annotated by S1 and S2 for these lesions are also included in S3’s masks, whereas the converse–pixels annotated by S3 overlapping with those of S1 and S2–is less frequent (Fig. 9b).

Across lesion types, segmentation-only variability is comparable in magnitude, with IoU values ranging from 0.40 for microaneurysms to 0.58 for cotton-wool spots (Fig. 9a). These differences largely reflect the average size of each lesion class: microaneurysms and exudates, which have the smallest diameters, also exhibit the lowest segmentation agreement–0.40 and 0.45, respectively–compared with 0.54 and 0.58 for hemorrhages and cotton-wool spots. Segmentation uncertainty primarily affects the boundary pixels, and for small lesions, these peripheral pixels constitute a large fraction of the entire mask, amplifying variability.

Furthermore, visual inspection of manual annotations shows that lesion boundaries are rarely aligned with visible edges or distinctive image features. Overall, manual segmentation by senior retina specialists appears inherently imprecise.

### Phase II: Interobserver variability with pre-segmentation

The second annotation phase aimed to quantify interobserver variability when lesion delineation was assisted by AI-generated pre-segmentation maps. Across the 16 images re-annotated by S1, J1, and J2 using this approach, the pixel-wise mIoU reached 0.71, a substantially higher agreement than that obtained among S1, S2, and S3 during the purely manual segmentation phase. Rule-based grading applied to those segmentation maps achieves a Gwet AC2 agreement coefficient of 0.95 (95% CI 0.89–1.00.89.00), indicating significantly higher inter-observer consistency compared to rule-based grading applied to purely manual annotation and even compared to visual grading (see Supplementary Material).

**Fig. 10:**
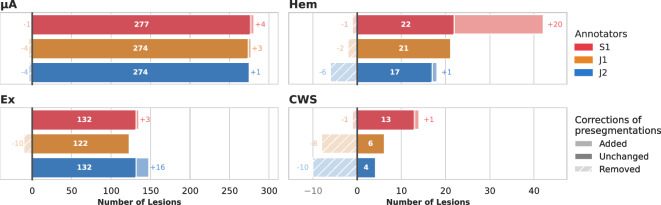
When correcting AI segmentation maps, 90% of lesions after human inspection are presegmented lesions which considerably reduces interobserver variability. Each bar summarizes the correction of the presegmentation maps per lesion types and per annotator. Only the *added* lesions were manually segmented by humans, the *unchanged* lesions come from the AI presegmentation and share the exact same shape accross annotators.

This marked reduction in interobserver variability reflects the high quality of the pre-segmentation masks, which required only limited manual adjustments. Indeed, 90% of the lesions present after correction originated directly from the pre-segmentations (Fig. 10). Only a small number of lesions were added manually: 8 microaneurysms (3% of the total), 1 cotton-wool spot, and 19 exudates (13%). Hemorrhages constitute an exception: whereas J1 added none and J2 added only one, S1 manually segmented 20 additional hemorrhages, effectively doubling their count relative to the AI pre-segmentation. This confirms the annotator-specific bias of S1 towards systematically labeling more hemorrhages. This bias identified in Phase I persisted in Phase II despite the availability of pre-segmentations. Regarding the false positives from the AI model, only 9% of pre-segmented lesions were removed by at least one annotator. This trend is consistent across all lesion types, except for cotton-wool spots: 13 of the 14 pre-segmented CWS (93%) were removed. This lesion type is the rarest, and its limited representation and diversity in the model’s training data undermine the reliability of CWS pre-segmentation.

Although manual corrections were overall infrequent, they were subject to the same detection variability characterized in Phase I. Among all deleted lesions, only 24% (9/37) were removed by more than one annotator, and none of the manually added lesions were jointly detected. Nevertheless, because these corrections were marginal compared to the large number of retained pre-segmented lesions, detection variability remained low overall. F1-scores were markedly higher than in purely manual annotation for both microaneurysms (F1 = 0.99) and exudates (F1 = 0.94). Agreement for hemorrhages (F1 = 0.68) also improved, although to a lesser extent due to the detection bias of S1, which introduced disagreements with J1 and J2. For cotton-wool spots–where pre-segmentation was unreliable–the detection agreement (F1 = 0.58) was close to that observed under purely manual segmentation (F1 = 0.40).

The segmentation-only component of interobserver variability was negligible under this pre-segmented annotation protocol. All jointly detected lesions were presegmented by the AI model, as every manually added lesion was unique to a single annotator. Moreover, among the pre-segmented lesions that were retained, only four underwent boundary corrections (two microaneurysms by J1 and two hemorrhages by J2); the remaining 444 lesions were left unchanged. Interestingly, the average diameters of manually added lesions (12 px for microaneurysms and 17 px for exudates) were consistent with a precise annotation style, similar to S1’s delineation behavior during manual segmentation and to the AI’s pre-segmentation output (mean diameters: 13 px and 20 px, respectively). This pattern was observed across all three annotators, suggesting that even in the absence of explicit guidelines the pre-segmentations acted as implicit instructions, shaping the annotators’ segmentation style.

## Discussion

This study quantifies the strong interobserver variability in lesion detection and segmentation. While such variability was expected, its magnitude had not been previously measured. The findings may appear concerning: segmentation agreement is low, and detection varies significantly across annotators. On average, more than one third of manually annotated lesions were annotated by one senior retinologist and not by either of the other two; when jointly detecting a lesion, they disagreed on half of the annotated pixels.

This level of disagreement far exceeds what has been reported in previous interobserver studies focused on anatomical structures such as the optic disk or retinal vessels. On vessels, Neimeijer et al. reported a Dice similarity score of 0.79 for the second annotator^21^; Kai et al. measured an interobserver Dice of 0.96 between senior and junior annotators^22^; on the optic disk, Kovalyk et al. reported an interobserver Dice of 0.83^23^; Kumar et al. measured Dice scores ranging from 0.97 to 0.98 between 5 annotators^24^. In all those cases, variability was largely attributed to differences in boundary delineation. However, lesions are inherently more fragmented, diverse in shape and size, and often subtle in appearance, making their annotation a technically demanding task. The interobserver variability resulting from their segmentation is plural and cannot be explained solely by boundary disagreements.

Interpreting the nature of this variability is essential for determining appropriate strategies to prevent, mitigate, or explicitly model it. In our view, disagreements between expert annotators fall into three categories. **(1)** *Annotation-style bias*: differences in granularity or implicit annotation rules yield segmentations that are each clinically acceptable but stylistically distinct. In such cases, each annotation style should be captured independently through style-aware or multi-style learning approaches^27,28^. **(2)** *Clinically meaningful uncertainty*: disagreements that reflect true ambiguity in lesion presence or boundaries. These cases call for multi-grader annotations^29^ or fuzzy or uncertainty-aware models (e.g., variational approximation using Monte-Carlo Dropout^30^ or probabilistic segmentation^31^). **(3)** *Random, non-informative noise*: imprecisions that do not reflect clinically relevant ambiguity and should be filtered out by the model through regularization or batching strategies.

In practice, most of the lesion segmentation literature bypasses this issue by training individual models on each dataset without accounting for annotation style or uncertainty. This has the unfortunate effect of tying each model’s style to its training dataset and lowering inter-dataset generalizability. Conversely, multi-dataset training may prevent developing a predictable model style. Both approaches overlook the multifactorial nature of interobserver variability in lesion annotation uncovered in this work. In particular, two components must be considered separately: detection variability and boundary variability.

Detection variability reflects both systematic annotation biases and intrinsic uncertainty. Differences in the number of lesions identified across annotators primarily indicate interobserver bias in annotation exhaustiveness. Although such biases could likely be mitigated through standardized annotation guidelines, they reflect real-world annotation campaigns and tele-ophthalmology screening programs, which often operate without detailed segmentation protocols. Beyond these systematic effects, lesion detection is also affected by genuine uncertainty, particularly for low-contrast lesions such as cotton-wool spots or peripheral findings degraded by illumination artifacts. Our findings also reveal substantial ambiguity in distinguishing small red lesions as microaneurysms or hemorrhages. This challenge was independently highlighted by He et al.^32^, who reported a classification accuracy of 54%–near chance level–when differentiating small dot red lesions. Consistent with their recommendations, our results support merging these lesions into a single class when fundus photography is the only available option.

Regarding pure segmentation variability, all three natures of variability coexist. Differences in annotation granularity clearly indicate a stylistic bias–ranging from the fine delineation of each lesion to the coarser labeling of pathological regions. This issue extends beyond our dataset: publicly available benchmarks exhibit diverse–and sometimes inconsistent–segmentation styles. Retinal Lesions^17^ delineate broad pathological areas; IDRiD^14^ and DDR^15^ annotate each lesion individually; in FGADR^16^, the segmentation style varies across images. No consensus exists on the optimal style for training segmentation models: should annotation favor speed and quantity, or precision and rarity? Beyond stylistic differences, variability in boundary placement reflects genuine uncertainty for diffuse lesions (hemorrhages, CWS), while small shifts in lesion position likely represent non-informative noise. Indeed, although senior retinologists are highly experienced in diagnosis, they are not trained to produce pixel-accurate segmentation masks–making some degree of imprecision unavoidable.

From these observations, several recommendations emerge. First, regarding annotation protocols: **(1)** Annotation tools can introduce biases; default brush settings and zoom levels influence the granularity of annotations and should be chosen carefully. **(2)** Image pre-processing can mitigate annotation deterioration due to uneven illumination, but aggressive color transformation may induce false positives; further work is needed to assess their impact on lesion detection and DR grading. **(3)** Pre-segmentation substantially reduces interobserver variability, but its effectiveness depends on model quality: manually added lesions remain subject to the same segmentation uncertainties and positional imprecision described earlier. Semi-automated tools (e.g., MedSAM) that align manually added contours to nearby image structures may further reduce variability. More generally, because manual boundaries are inherently imprecise, refinement via such tools is preferable. **(4)** Annotation guidelines should explicitly define the expected granularity; as shown by Radsch *et al.*^33^, example images are often more effective than textual instructions. Pre-segmentation masks may serve this role.

Second, regarding the definition of standard DR screening protocols based on lesion counts: **(1)** Microaneurysms are not reliably distinguishable from small hemorrhages on fundus imaging; they should be grouped into a single category, with lesion size serving as the primary determinant of severity. This approach aligns with the American Academy of Ophthalmology’s Preferred Practice Pattern, which, since 2016, no longer distinguishes microaneurysms from small dot hemorrhages. **(2)** For a given annotator, systematic over-detection of lesions does not necessarily translate into an overestimation of DR severity. This stands in contrast to the rigidity of grading guidelines, which rely heavily on lesion counts. Paradoxically, despite the large variability in lesion-level annotations, experts often reach higher agreement on overall DR severity–suggesting that clinical decision-making incorporates additional implicit or contextual cues beyond formal criteria. **(3)** The distinction between mild, moderate, and severe DR relies predominantly on hemorrhage counts; yet, hemorrhages show particularly high detection variability. A more nuanced representation of DR severity may therefore be needed, potentially shifting from discrete grades toward a continuous or multi-dimensional severity scale.

Third, regarding the use of manually annotated ground truths for model training and evaluation: **(1) **Because manually segmented lesion contours and localizations are unreliable, model evaluation should complement pixel-wise metrics with detection metrics such as the tailored precision, recall, and F1-score proposed in this paper. Using IoU as the primary metric for comparing lesion segmentation algorithms should be reconsidered: this metric is too sensitive to segmentation variability, which we have demonstrated to be elevated and clinically irrelevant for diagnosing DR. **(2) ** Given the substantial stylistic variability across datasets and annotators, training with pixel-wise losses will irremediably lead to overfitting the annotation style of the training set and impair the model’s generalization capacities. In this context, we suggest either using multi-style learning architectures that enable control over the desired segmentation style at inference, or learning to detect lesions instead of segmenting them, for example, by regressing the lesions center points. (3) Purely rule-based algorithms that strictly apply diagnostic guidelines to lesion segmentation maps will likely perform poorly because they do not capture the implicit clinical cues that we have shown are important. Therefore, contextualization of the segmentation maps is necessary, for example, through the construction of a graph of pathological structures^34^. **(4) **The confusion between microaneurysms and dust artifacts remains underexplored. Additional work is needed to detect artifacts–either through the experimental trick noted earlier or through synthetic generation.

This study was initially intended as a simple validation of the MAPLES-DR annotations. In the absence of prior work on annotation variability for retinal pathological structures, we designed the experimental protocol by drawing on comparable studies focusing on anatomical structures. In hindsight, however, this protocol was insufficient to precisely quantify the high variability specific to retinal lesions. Four aspects, in particular, limit its scope. **(1)** By focusing on low-severity DR images, we likely overestimate the interobserver variability measured in detecting severe lesions (Hem, Ex, and CWS). For these lesions, detection agreement is indeed higher in patients with advanced DR. More broadly, the limited sample size (51 images and 3 annotators) reduces the precision of the variability estimates, which should therefore be interpreted in terms of order of magnitude rather than exact numerical values. **(2)** Intraobserver variability was not assessed in our experimental protocol. An indirect estimate–based on comparing annotations produced by the same retinologist for MAPLES-DR and for the present study–suggests that it is non-negligible and of a similar magnitude to interobserver variability. Consequently, while our findings suggest that variability in lesion detection and segmentation stems from annotator-specific biases and intrinsic uncertainty, a dedicated intraobserver study is necessary to quantify the respective contributions of these two sources of variability. **(3)** The change in annotators between phase I and phase II tempers the conclusion that AI-based pre-segmentation improved inter-annotator consistency. Although the difference in interobserver variability measured with and without pre-segmentations leaves little doubt about their beneficial impact, part of the observed improvement could also stem from the replacement of annotators, particularly given that the phase II annotators were less experienced. Additionally, our study does not evaluate biases introduced by pre-segmentation. We know from our experience annotating MAPLES-DR that the quality of the pre-segmentation model is critical for effectively reducing interobserver variability by requiring fewer manual corrections. However, we did not evaluate the natural–and potentially excessive–trust that annotators place in the AI output. Addressing this issue would require a separate study in which several versions of pre-segmentation are presented to annotators, and their corrections are compared. **(4)** The absence of a standardized annotation protocol–including detailed instructions on expected annotation styles, preprocessing, zoom levels, and monitor resolutions–allowed us to identify multiple sources of variability in annotations and determine an upper bound of interobserver variability. However, this prevented us from isolating the effects of each source. A more thorough, factor-wise investigation would help refine the proposed guidelines for reliable lesion annotation.

In summary, this study is, to our knowledge, the first to characterize lesion annotation variability in fundus photography. It uncovers an unexpectedly high level of variability and identifies their primary contributing factors. These findings support the informed use of these annotations in machine-learning settings and in clinical DR screening, and, on a more fundamental level, they establish the foundation for subsequent studies necessary to quantify the relative impact of the variability confounders and to develop a reliable protocol for annotating retinal lesions.

## Conclusion

We showed that manual annotation of lesions on fundus images exhibits substantial interobserver variability and provided an overview of its main components. Lesion detection is affected by both bias and disagreement: 38% of the lesions identified by one annotator were not identified by either of the other two, with small red lesions particularly impacted. These small lesions frequently lead to inconsistent classification between microaneurysms and hemorrhages, with more than half being labeled differently across annotators. Lesion localization was also imprecise, with a typical positional offset of about one-third of the lesion diameter. Contour delineation proved similarly unreliable, with one in two pixels differing between two segmentations of the same lesion. Despite this substantial variability at the segmentation level, its impact diminishes when establishing diabetic retinopathy severity, although grading is formally defined by lesion counts. This suggests that clinicians, in practice, may rely on implicit criteria through a more holistic or experience-based assessment.

Finally, compared with full manual annotation, editing pre-segmented maps substantially reduces interobserver variability. Future work will examine how semi-automated segmentation tools may further decrease variability and support more consistent annotation workflows.

## Supplementary Information

Supplementary Information.

## Data Availability

All the lesion segmentation maps studied in this research—manual and semi-manual annotations, as well as presegmentation maps—were deposited in the MAPLES-DR database repository and are available at the following URL: 10.6084/m9.figshare.24328660.
