# Supplementary material for: Retinal lesion annotation on fundus imaging: an interobserver variability study
Source: Sci Rep. 2026 May 28;16:25020. doi: 10.1038/s41598-026-53558-5 (PMC13462781; doi:10.1038/s41598-026-53558-5)
Supplement: Supplementary file 1 [file 41598_2026_53558_MOESM1_ESM.pdf]

Supplementary Materials

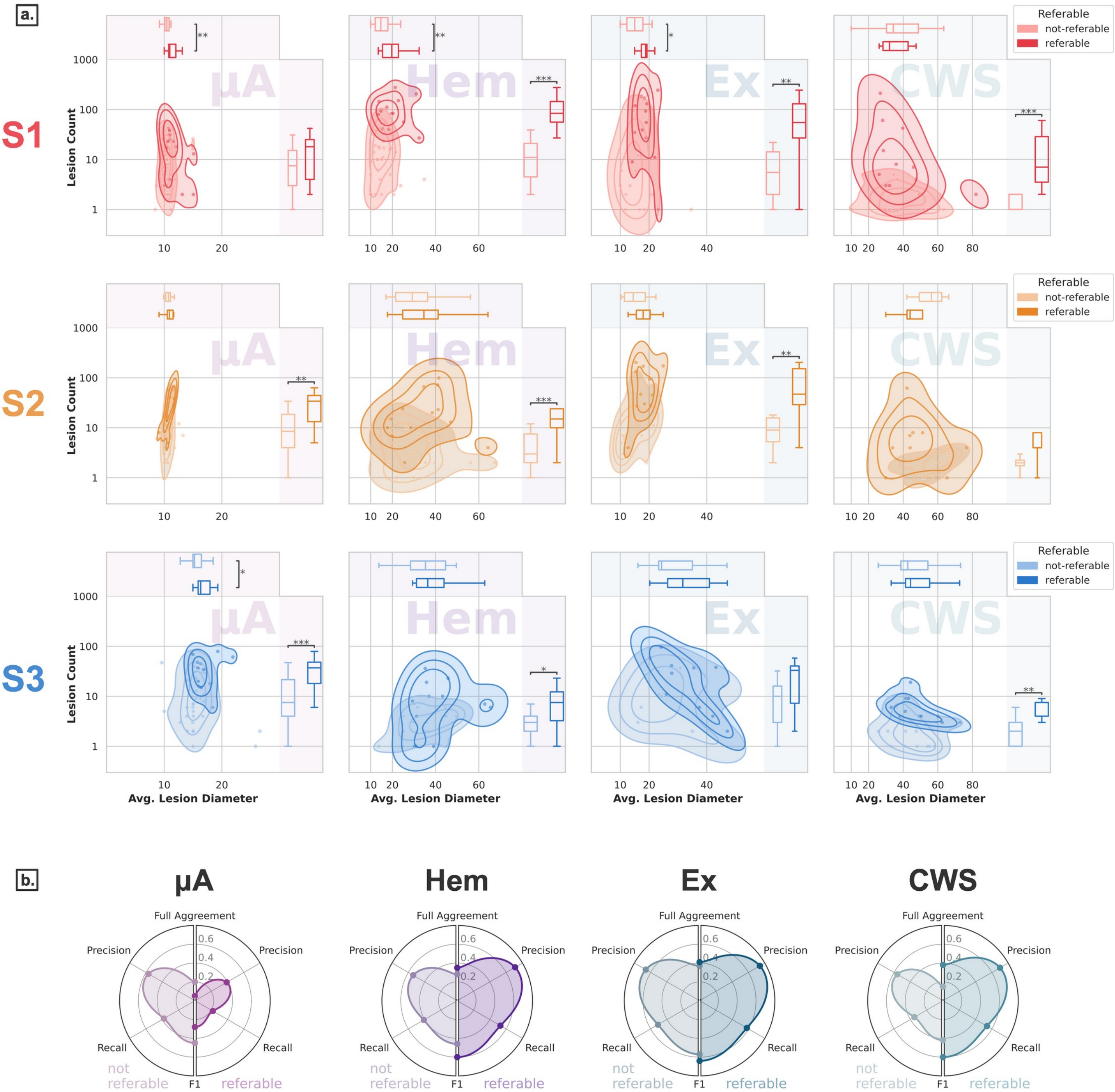

Figure S1. Severe lesions (Hem, Ex and CWS) are more numerous and detected more consistently in patients with advanced DR. Conversely, µA detection consistency is higher for patients with mild DR. **(a)** Distributions of the lesion count and average diameter per image by annotator and lesion type for not-referable images (healthy and mild DR, n=38) and referable images (moderate and severe DR, n=13). Significance is measured using a two-sided Mann–Whitney U-test (*: $p < 0.05$; **: $p < 0.01$; ***: $p < 0.001$). **(b)** Average interobserver agreement metrics for lesion detection comparing not-referable with referable images.

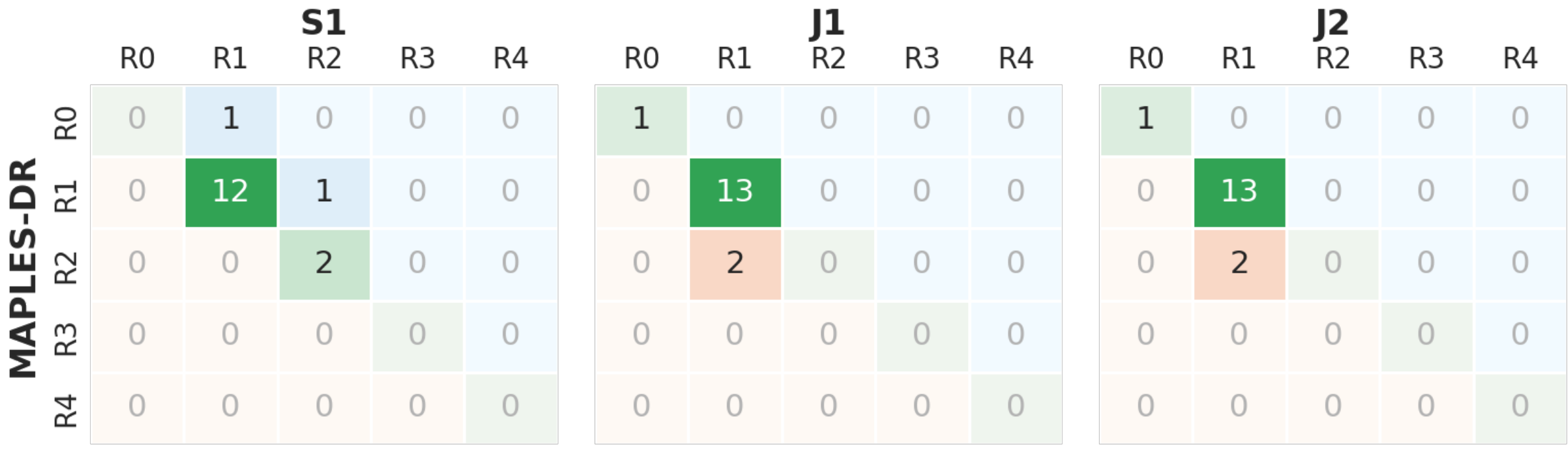

Figure S2. Rule-based DR grades computed from IA-assisted lesions annotation and compared to MAPLES-DR ground-truth labels.
